# African‐origin protective haplotype for Alzheimer's disease in *APOEε4* carriers

**DOI:** 10.1002/alz70855_104916

**Published:** 2025-12-24

**Authors:** Luciana Bertholim Nasciben, Derek Van Booven, Wanying Xu, Liyong Wang, Sofia Moura, Aura M Ramirez, Karen Nuytemans, Derek M. Dykxhoorn, Farid Rajabli, William K. Scott, David A. Davis, Regina T Vontell, Katalina F. McInerney, Michael L Cuccaro, Goldie S Byrd, Jonathan L Haines, Larry D Adams, Margaret Pericak‐Vance, Juan I Young, Fulai Jin, Anthony J Griswold, Jeffery M Vance, ADSP (Alzheimer's Disease Sequencing Project)

**Affiliations:** ^1^ University of Miami Miller School of Medicine, Miami, FL, USA; ^2^ John P. Hussman Institute for Human Genomics, University of Miami Miller School of Medicine, Miami, FL, USA; ^3^ Department of Genetics and Genome Sciences, School of Medicine, Case Western Reserve University, Cleveland, OH, USA; ^4^ Dr. John T. Macdonald Foundation Department of Human Genetics, University of Miami Miller School of Medicine, Miami, FL, USA; ^5^ Brain Endowment Bank, Department of Neurology, University of Miami Miller School of Medicine, Miami, FL, USA; ^6^ Department of Neurology, University of Miami Miller School of Medicine, Miami, FL, USA; ^7^ Wake Forest University School of Medicine, Winston‐Salem, NC, USA; ^8^ Cleveland Institute for Computational Biology, Department of Population & Quantitative Health Sciences, School of Medicine, Case Western Reserve University, Cleveland, OH, USA

## Abstract

**Background:**

We have reported a statistical interaction between the African ancestry specific A allele at rs10423769 and *APOEε4*, where the presence of the A allele is associated with a reduction in AD risk up to 75% in *APOEε4* homozygotes. The mechanism by which this variant confers protection could provide insights into new therapeutics for *APOEε4* carriers. However, the genomic context of this protective variant is complex with a high number of segmental duplications (SD) and structural variants (SVs). Thus, we have used advanced genomic techniques to investigate the region of ∼2MB between rs10423769_A and *APOE* to gain further insights into potential AD‐protective mechanisms.

**Method:**

We used high‐coverage (90x) PacBio HiFi whole genome sequencing on 9 samples of rs10423769_A/A, rs10423769_A/G, and rs10423769_G/G carriers. We performed genome assembly with hifiasm to identify contigs spanning the whole region between the protective allele and *APOE*. We compared the contigs using minigraph and Bandage. We performed Hi‐C sequencing from 13 brain autopsy samples (seven rs10423769_A carriers) and used in‐house bioinformatics tools (enhanced Hi‐C Capture Analysis ‐ eHiCA) to identify chromatin loops indicative of potential interactions between the protective and *APOE* loci.

**Result:**

Long read sequencing detected the co‐occurrence of an expanded VNTR containing multiple MEF2D transcription factor binding sites within the protective haplotype. A higher number of SVs in the SD region surrounding rs10423769_A haplotype were detected when compared to the reference and are under investigation. eHiCA using rs10423769 or the *APOE* promoter as the bait suggested that rs10423769 locus and the *APOE* promoter region have evidence of long‐range, physical interactions. Furthermore, both rs10423769 locus and the *APOE* promoter interact with common regions in between the two baits, including a cluster of zinc finger (*ZNF*) genes upstream of *APOE*. The reciprocal eHiCAs suggest high confidence interactions between these loci and with other regions of chr19.

**Conclusion:**

Our results show that the protective haplotype locus of African origin has long‐range, physical interactions with the *APOE* promoter and other regions on chr19. It also carries an expanded VNTR specific to the protective haplotype containing multiple transcription factor binding sites.

